# Predictive functional assay‐based classification of PMS2 variants in Lynch syndrome

**DOI:** 10.1002/humu.24387

**Published:** 2022-04-28

**Authors:** Emily Rayner, Yvonne Tiersma, Cristina Fortuno, Sandrine van Hees‐Stuivenberg, Mark Drost, Bryony Thompson, Amanda B. Spurdle, Niels de Wind

**Affiliations:** ^1^ Department of Human Genetics Leiden University Medical Center Leiden the Netherlands; ^2^ Princess Maxima Center for Child Oncology Utrecht the Netherlands; ^3^ QIMR Berghofer Medical Research Institute Brisbane Queensland Australia; ^4^ Department of Clinical Genetics Erasmus Medical Center Rotterdam the Netherlands; ^5^ Department of Pathology Royal Melbourne Hospital Parkville Victoria Australia

**Keywords:** diagnostic assessment, DNA mismatch repair, functional analysis‐based classification, Lynch syndrome, PMS2, variants of uncertain significance

## Abstract

The large majority of germline alterations identified in the DNA mismatch repair (MMR) gene *PMS2*, a low‐penetrance gene for the cancer predisposition Lynch syndrome, represent variants of uncertain significance (VUS). The inability to classify most VUS interferes with personalized healthcare. The complete in vitro MMR activity (CIMRA) assay, that only requires sequence information on the VUS, provides a functional analysis‐based quantitative tool to improve the classification of VUS in MMR proteins. To derive a formula that translates CIMRA assay results into the odds of pathogenicity (OddsPath) for VUS in PMS2 we used a set of clinically classified PMS2 variants supplemented by inactivating variants that were generated by an in cellulo genetic screen, as proxies for cancer‐predisposing variants. Validation of this OddsPath revealed high predictive values for benign and predisposing PMS2 VUS. We conclude that the OddsPath provides an integral metric that, following the other, higher penetrance, MMR proteins MSH2, MSH6 and MLH1 can be incorporated as strong evidence type into the upcoming criteria for MMR gene VUS classification of the American College of Medical Genetics and Genomics and the Association for Molecular Pathology (ACMG/AMP).

## Graphical abstract

Three‐step classification of Lynch syndrome‐associated Variants of Uncertain Significance (VUSs) in any of the DNA mismatch repair genes. (1) Protein VUSs are synthesized in vitro, based on sequence information provided by the clinical geneticist. (2) Their functional activity are assayed and quantified. (3) The odds of pathogenicity are calculated and incorporated in the final classification according to the American College of Medical Genetics and Genomics and the Association for Molecular Pathology BS3/PS3 criteria

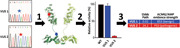

## 1 INTRODUCTION

Lynch syndrome (LS; MIM# 120435) is an autosomal dominant cancer predisposition, caused by a heterozygous inactivating germline defect in one of the major mismatch repair (MMR) genes: *MLH1* (MIM# 120436), *MSH2* (MIM# 609309), *MSH6* (MIM# 600678), or *PMS2* (MIM# 600259). Inadvertent inactivation of the wild‐type allele in somatic cells results in MMR deficiency, which reconfers a mutator phenotype that predisposes to LS‐associated colorectal, endometrial, and other visceral cancers (Cerretelli et al., 2020). Of note, since a heterozygous MMR‐inactivating gene defect is not (directly) disease‐causing, nor is complete MMR‐deficiency, we propose to use the term “predisposing,” rather than “pathogenic” for MMR‐inactivating germline defects.

MMR gene variants are classified using criteria that are based on guidelines provided by the International Society for Gastrointestinal Hereditary Tumors (InSiGHT) Variant Interpretation Committee (VIC) and the American College of Medical Genetics and Genomics and the Association for Molecular Pathology (ACMG/AMP) (Richards et al., 2015; B. A. Thompson et al., 2014). These criteria include allele frequency, cosegregation, age of tumor onset, microsatellite instability (MSI), and loss of protein staining in tumors, among others. Predisposing variants in PMS2 confer a low prevalence of cancer due to the protein's genetic redundancy (11%–20% lifetime colorectal cancer risk by age 70) (Senter et al., 2008; ten Broeke et al., 2015). The current clinically focused approach of variant classification, therefore, is often underpowered to classify PMS2 VUS, and 98% of PMS2 missense variants (1490/1521) remain unclassified (Class 3, VUS) in the ClinVar database (Accessed, August 19, 2021) (Landrum et al., 2018). Consequently, personalized healthcare for carriers and affected relatives cannot be implemented. For this reason, it is of the utmost importance to classify these VUS.

**Figure 1:**
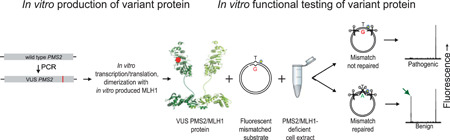
Flow diagram of the complete in vitro mismatch repair activity (CIMRA) assay. The variant of interest is produced by a PCR procedure, expressed in a coupled transcription‐translation kit and then heterodimerized with MLH1 protein, expressed from wild‐type MLH1 cDNA. Then the functional activity of the variant heterodimer is assessed in a complementation assay containing a substrate containing a T.G mismatch with a linked fluorescent group (star), and MLH1/PMS2‐deficient cell extract. Repair of the mismatch recreates a Hin1II‐cleavable restriction endonuclease site, resulting in the generation of a fluorescent diagnostic fragment (green arrow) that is visualized by capillary electrophoresis. Following calibration of the assay results against independently classified variants, the relative abundance of this diagnostic fragment is translated into an odds of pathogenicity for the variant PMS2/MLH1 heterodimer (Drost, Koppejan, et al., 2013; Drost, Lützen, et al., 2013).

Since loss of biochemical MMR function is causative of cancer predisposition in LS, functional assays can be used to improve the classification of VUS. We have developed the complete in vitro mismatch repair activity (CIMRA) assay that rapidly quantifies the MMR activity of cDNA‐encoded MMR protein VUS in vitro, based on sequence information of the VUS alone (Figure 1; Drost, Koppejan, et al., 2013; Drost, Lützen, et al., 2013). We have previously calibrated and validated the CIMRA assay for MSH2, MSH6, and MLH1 protein VUS (Drost et al., 2019, 2020). This was done by regression of the assay output against variants, independently classified using clinical data. In case there were insufficient clinically classified predisposing variants available we included MMR‐inactivating variants produced by a genetic screen, as a proxy for such variants. The OddsPath may be combined with a probability of pathogenicity based on computational predictions (Prior‐P) to calculate a posterior probability of pathogenicity (Posterior‐P) between 0 and 1 (B. A. Thompson et al., 2013). This approach has been proven to have high sensitivities and specificities for both predisposing and benign variants of the MSH2, MSH6, and MLH1 MMR proteins (Drost et al., 2019, 2020).

Here, we have used a set comprised of clinically classified benign and predisposing PMS2 substitution variants, supplemented with functionally disrupting (as a proxy for predisposing) Pms2 variants, generated using a genetic screen, to calibrate and validate the output from the CIMRA assay for PMS2 VUS into an OddsPath. We demonstrate that the OddsPath is a robust metric that can be incorporated in the ACMG/AMP guidelines for PMS2 VUS classification.

## 2 METHODS

### 2.1 Cell culture and generation of *Pms2*‐hemizygous mouse embryonic stem cells using CRISPR‐Cas9

Hemizygous *Pms2* mouse embryonic stem cells (mESCs) for use in the genetic screen were generated using CRISPR‐Cas9. Briefly, two pairs of single‐stranded oligonucleotides with BbsI overhangs targeting the C‐terminal and N‐terminal end of *Pms2* were annealed and cloned into the Cas9 vector PX330‐Puro (Cong et al., 2013). mESCs were transfected with the two PX330‐Puro plasmids containing the gRNA's using Lipofectamine 2000 (Thermo scientific) and after 24 h, positively transfected cells were selected with 1 μg/ml Puromycin. After selection, cells were subcloned, and *Pms2* hemizygosity was confirmed using allele‐specific PCR. Pms2 protein expression was checked by immunoblotting.

### 2.2 Generation of a set of pathogenic PMS2 missense variants using a genetic screen

**Figure 2:**
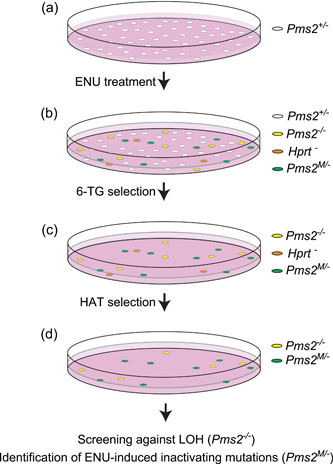
A genetic screen to identify inactivating variants in Pms2. Experimental steps to generate Pms2‐inactivating variants in mouse embryonic stem cells (mESCs), to be used as a proxy for predisposing variants. (a) A Pms2‐heterozygous (Pms2^+/−^) cell line is treated with N‐ethyl‐N‐nitrosourea to induce random substitution mutations. (b) Cells that have undergone loss of heterozygosity (LOH) at Pms2 (LOH, Pms2^−/−^), cells with an inactivating mutation in Hprt (Hprt^−/−^), and cells that have acquired an inactivating mutation in the wild‐type allele of Pms2 (Pms2M/−) are selected by their tolerance to 6‐TG. (c) Cells with an Hprt‐inactivating mutation are eliminated by culture in hypoxanthine‐aminopterin‐thymidine (HAT)‐supplemented medium. (d) The inactivating missense substitutions in the remaining clones without LOH (LOH clones are screened against by intragenic Pms2 PCR) are identified by cDNA sequence analysis. These inactivating substitution variants are subsequently included in the calibration and validation set.

To compile a set of inactivating amino acid substitutions in Pms2, as a proxy for cancer‐predisposing variants, a genetic screen was performed, essentially as described for *Msh2* and *Msh6* (Drost, Lützen, et al., 2013). In brief, mESCs hemizygous for *Pms2* were exposed to the strong mutagen N‐ethyl‐N‐nitrosourea (ENU; Sigma‐Aldrich) with the aim of introducing random missense substitutions in the genome (Figure 2). Cells that had become MMR‐deficient, presumably by an inactivating mutation at the monoallelic *Pms2* gene, were selected using the Guanine analog 6‐Thioguanine (6‐TG, Sigma‐Aldrich). 6‐TG is cytotoxic via the lethal processing of methyl‐6‐thioGuanine:Thymidine mismatches by MMR. Consequently, a defect in MMR results in survival to the drug and 6‐TG is a widely used agent used to select for MMR‐deficient cells and to test for functional inactivation and cancer predisposition caused by a variant (Drost et al., 2020; Houlleberghs et al., 2016; Houlleberghs et al., 2020). Surviving clones were treated for 1 week in hypoxanthine‐aminopterin‐thymidine (HAT)‐supplemented medium (Gibco) to kill inadvertent *Hprt*‐mutant cells that also survive 6‐TG treatment. Next, surviving clones were picked and expanded in 96‐well plates. These clones were screened against loss of heterozygosity (LOH) at *Pms2*, an alternative way of losing the wild‐type *Pms2* allele, by intragenic allele‐specific PCR. From remaining clones, *Pms2* cDNA was generated and the critical, conserved, domains were sequenced using Sanger sequencing to identify the inactivating amino acid substitution. All primer sequences and PCR protocols are available upon request.

### 2.3 Selection of missense substitutions for CIMRA assay calibration and validation

We derived a set of PMS2 missense variants from the ClinVar and InSiGHT databases: 20 had been classified as IARC Class 1/2 [(likely) benign], and 11 as Class 4/5 [(likely) pathogenic], based on only clinical data (Accessed, October 28, 2019) (Supporting Information: Table S1). A total of 31 unique inactivating missense substitutions were derived from the genetic screen (Supporting Information: Table S2). A part of the variants identified in the databases and screen were excluded from the calibration and validation set based on the following criteria: missense variants in the start codon of PMS2; proven spliceogenic variants; variants with an unavailable in silico prior probability (Prior‐P); nonconserved amino acid variants (if derived from the murine genetic screen); one variant used as a predisposing control in the CIMRA assay (Supporting Information: Tables S1 and S2).

### 2.4 Complete in vitro MMR activity (CIMRA) assay

CIMRA assays of the PMS2 missense variants were performed essentially as previously described (Drost, Koppejan, et al., 2013). Briefly, the variant *PMS2* cDNA is synthesized by mutagenic PCR followed by in vitro transcription and translation of the variant PMS2 protein as well as of the wild‐type MLH1 dimerization partner. Nuclear extract from *MLH1*‐deficient human HeLa cells (which is also deficient for PMS2 protein due to protein instability without its partner) generated by CRISPR‐Cas9 gene targeting is complemented with in vitro produced human (variant) PMS2/MLH1 protein and a mismatch‐containing fluorescent substrate. Repair of the mismatch recreates a Hin1II restriction site, which is quantified using capillary fluorescent gelectrophoresis. Repair deficiency resulting from an inactivating VUS causes the absence of the repair‐diagnostic fluorescent fragment, indicating that the VUS is cancer‐predisposing.

### 2.5 Regression for CIMRA assay calibration and validation

Regression of CIMRA assay results against the known or assumed statistical probability of pathogenicity of the variants from the test set was performed following methods similar to those published for MLH1, MSH2, and MSH6 variants (Drost et al., 2019). Given the relatively small sample size of the PMS2 variant set (N = 51), 10‐fold cross‐validation was chosen as the preferred method to assess the fit of the regression model; 80% of the data are considered the training set and 20% of the data is considered the validation set in 10 different versions of the model. Analysis was performed using the Caret package in RStudio version 1.0.153. Only five of these variants had information on pathogenicity in the InSiGHT database based on multifactorial likelihood analysis (Supporting Information: Table S1) (B. A. Thompson et al., 2014). For this reason, variants assigned to Class 4 (likely pathogenic) using clinical criteria and the presumed inactivating variants derived from the genetic screen were conservatively assigned a probability of pathogenicity of 0.95 (Class 4), while variants in Classes 1 and 2 [termed (likely) benign]) were conservatively assigned a probability of pathogenicity of 0.05 (Class 2). These probabilities were then transformed to OddsPath using the following formula (B. Thompson et al., 2021): (Probability)/(1‐Probability), and used as the dependent variable in the form of log10 in the regression formula. The relative average of the CIMRA assay results for each variant was used as the independent variable. To assess the fit of the regression model, a 10‐fold cross‐validation procedure was applied. A leave‐one‐out procedure was then used to calculate the OddsPath for each variant used for calibration of the regression formula. OddsPath for individual variants was aligned to ACMG/AMP evidence code strengths as per Tavtigian et al. (2018), shown in Supporting Information: Table S3.

## 3 RESULTS

### 3.1 Genetic screen for the identification of inactivating Pms2 missense variants

**Figure 3:**
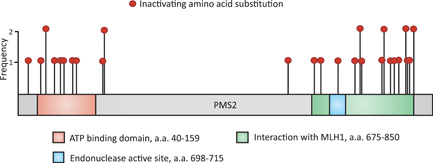
Frequency and location of genetic screen‐derived inactivating variants conserved between murine Pms2 and human PMS2A schematic representation of the PMS2 protein showing conserved ATP binding and MLH1 interactions domains and the endonuclease active site. The position and frequency of Pms2‐inactivating mutations (in red) identified in the genetic screen are plotted on the schematic.

To enable calibration of CIMRA activity of PMS2 variants into an OddsPath and its validation, a set of clinically classified Class 1/2 [(likely)benign] and Class 4/5 [(likely) pathogenic] variants was derived from the ClinVar and InSiGHT databases (Supporting Information: Table S1). Of the variants classified as (likely) pathogenic in ClinVar, five disrupted the start codon and one was used as the inactivating control in the CIMRA assay (p. Ser46Ile), and therefore these could not be used for calibration of the assay. Two variants (p. Ile668Val, p. Lys301Asn) were excluded since they have previously been shown to impact splicing (Li et al., 2015; B. A. Thompson et al., 2020; van der Klift et al., 2015). After these exclusion criteria, as described in Section 2, were applied, 20 (likely) benign and 3 (likely) predisposing missense PMS2 variants were included from the databases (Table 1). Unfortunately, presumably owing to the low cancer penetrance of inactivating PMS2 defects, too few clinically classified (likely) predisposing variants were available to generate a reference set for calibration purposes. To obtain sufficient functionally inactivating PMS2 substitution variants for assay calibration and validation we performed a genetic screen in mESc, as previously described for *Msh2* and *Msh6* (Drost et al., 2020; Drost, Lützen, et al., 2013; Figure 2). A total of 34 MMR‐deficient mESC lines were obtained from the genetic screen, containing 31 unique Pms2‐inactivating amino acid substitutions, to serve as proxy pathogenic/predisposing variants for calibration and validation (Supporting Information: Figure S1 and Table S2). Of these unique amino acid substitutions, two residues were not conserved between mice and humans and for one no Prior‐P could be derived as two nucleotides were altered. The human equivalents of the remaining 28 Pms2‐inactivating substitution variants were included, as proxies for predisposing PMS2 variants, in the calibration and validation set of the CIMRA assay (Figure 3, Table 1). Of note, variant p.Asn749Lys, identified in the genetic screen, was also listed as an unclassified human VUS in the ClinVar database (Accessed, October 15, 2021), which supports its role in cancer predisposition.

**Table 1:** CIMRA assay results and classification of substitution variants used for calibration and validation of the assay.

Source	Variant		CIMRA assay values			
	protein^b^	Prior‐P^c^	CIMRA assay activity^d^	OddsPath^e^	ACMG/AMP evidence strength^f^	Classification in database^g^
GS^a^	p.Ser28Phe	0.881	10.7	14.29	PS3—Moderate	NA
GS	p.Asp48Val	0.891	5.8	18.84	PS3—Strong	NA
GS	p.Leu56Pro	0.829	12.4	12.99	PS3—Moderate	NA
GS	p.Leu56Gln	0.766	7.7	16.92	PS3—Moderate	NA
GS	p.Gly74Glu	0.959	5.8	18.84	PS3—Strong	NA
GS	p.Thr89Ala	0.965	8.6	16.09	PS3—Moderate	NA
GS	p.Ile92Asn	0.901	10.3	14.61	PS3—Moderate	NA
GS	p.Leu111Pro	0.961	6.1	18.52	PS3—Moderate	NA
GS	p.Val168Glu	0.949	5.8	18.84	PS3—Strong	NA
GS	p.Arg169Ser	0.878	6.9	17.70	PS3—Moderate	NA
GS	P.Arg169His	0.539	4.6	20.16	PS3—Strong	NA
GS	p.Leu618Pro	0.708	29.3	5.05	PS3—Moderate	NA
GS	p.Gly680Asp	0.960	4.6	20.16	PS3—Strong	NA
GS	p.Lys690Glu	0.862	41.7	2.54	PS3—Supporting	NA
GS	p.Tyr707Asn	0.832	13.7	12.07	PS3—Moderate	NA
GS	p. Asn742Ile	0.940	4.9	19.83	PS3—Strong	NA
GS	p. Asn749Lys	0.916	6.2	18.42	PS3—Moderate	3
GS	p.Phe751Ser	0.857	3.8	21.10	PS3—Strong	NA
GS	p.Cys812Gly	0.780	4.4	20.39	PS3—Strong	NA
GS	p.Ser815Pro	0.967	8.7	12.25	PS3—Moderate	NA
GS	p.Leu822Pro	0.961	4.0	20.86	PS3—Strong	NA
GS	p.Ile831Ser	0.069	2.8	22.31	PS3—Strong	NA
GS	p.Met834Thr	0.918	3.9	20.98	PS3—Strong	NA
GS	p.Pro844His	0.854	3.9	20.98	PS3—Strong	NA
GS	p.His845Arg	0.946	4.8	19.94	PS3—Strong	NA
GS	p. Arg847Trp	0.891	3.0	22.06	PS3—Strong	NA
GS	p.Met850Arg	0.608	3.5	21.44	PS3—Strong	NA
GS	p.Met850Lys	0.654	2.6	22.57	PS3—Strong	NA
Database	p.Ser6Thr	0.003	143.6	0.01	BS3—Strong	1
Database	p.Ile18Val	0.557	78.1	0.38	BS3—Supporting	2
Database	p. Arg20Gln	0.040	105.4	0.08	BS3—Moderate	1
Database	p. Asn45Thr	0.809	5.4	19.27	PS3—Strong	4
Database	p.Ser46Asn	0.742	15.4	10.97	PS3—Moderate	4
Database	p.Asp60Glu	0.000	120.5	0.04	BS3—Strong	2
Database	p.Val63Met	0.004	108.9	0.07	BS3—Moderate	2
Database	p. Gln100Arg	0.013	146.4	0.01	BS3—Strong	1
Database	P.Thr485Lys	0.001	131.5	0.02	BS3—Strong	1
Database	p.Thr511Ala	0.002	107.0	0.08	BS3—Moderate	1
Database	p.Thr511Met	0.001	102.9	0.10	BS3—Moderate	1
Database	p. Asp534Glu	0.000	94.9	0.15	BS3—Moderate	2
Database	p.Lys541Glu	0.002	100.1	0.11	BS3—Moderate	1
Database	p. Arg563Leu	0.001	118.3	0.04	BS3—Strong	2
Database	p.Asn570Asp	0.001	100.9	0.11	BS3—Moderate	2
Database	p.Leu571Ile	0.000	110.5	0.06	BS3—Moderate	2
Database	p.Ile590Val	0.002	104.1	0.09	BS3—Moderate	2
Database	p.Thr597Ser	0.000	119.1	0.04	BS3—Strong	1
Database	p.Met622Ile	0.004	123.4	0.03	BS3—Strong	1
Database	p.Asn692Ser	0.002	81.5	0.32	BS3—Supporting	2
Database	p.Asn775Ser	0.157	94.2	0.09	BS3—Moderate	1
Database	p.Ser815Leu	0.935	3.9	20.98	PS3—Strong	4
Database	p.Gly857Ala	0.002	96.5	0.14	BS3—Moderate	1

Abbreviations: ACMG/AMP, American College of Medical Genetics and Genomics and the Association for Molecular Pathology; CIMRA, complete in vitro mismatch repair activity; NA, not available; OddsPath, odds of pathogenicity.

^a^ GS, genetic screen‐derived.

^b^ Amino acid numbering is based on the cDNA with +1 corresponding to the translation initiation codon in the GenBank reference sequence. PMS2: NP_000526.2.

^c^ In silico prior probabilities as previously calculated (B. A. Thompson et al., 2013).

^d^ Mean of CIMRA assay relative repair as determined experimentally during this project from at least 3independent replicates

^e^ Odds of pathogenicity for each variant resulting from the leave‐one‐out method.

^f^ Categorization of the variant using the OddsPath based on ACMG/AMP PS3/BS3 framework (https://www.insight-group.org/criteria). PS3 = Pathogenic, BS3 = Benign.

^g^ IARC classification for each variant as classified by the InSiGHT Variant Interpretation Committee (B. Thompson et al., 2014).

### 3.2 CIMRA assay calibration and validation

**Figure 4:**
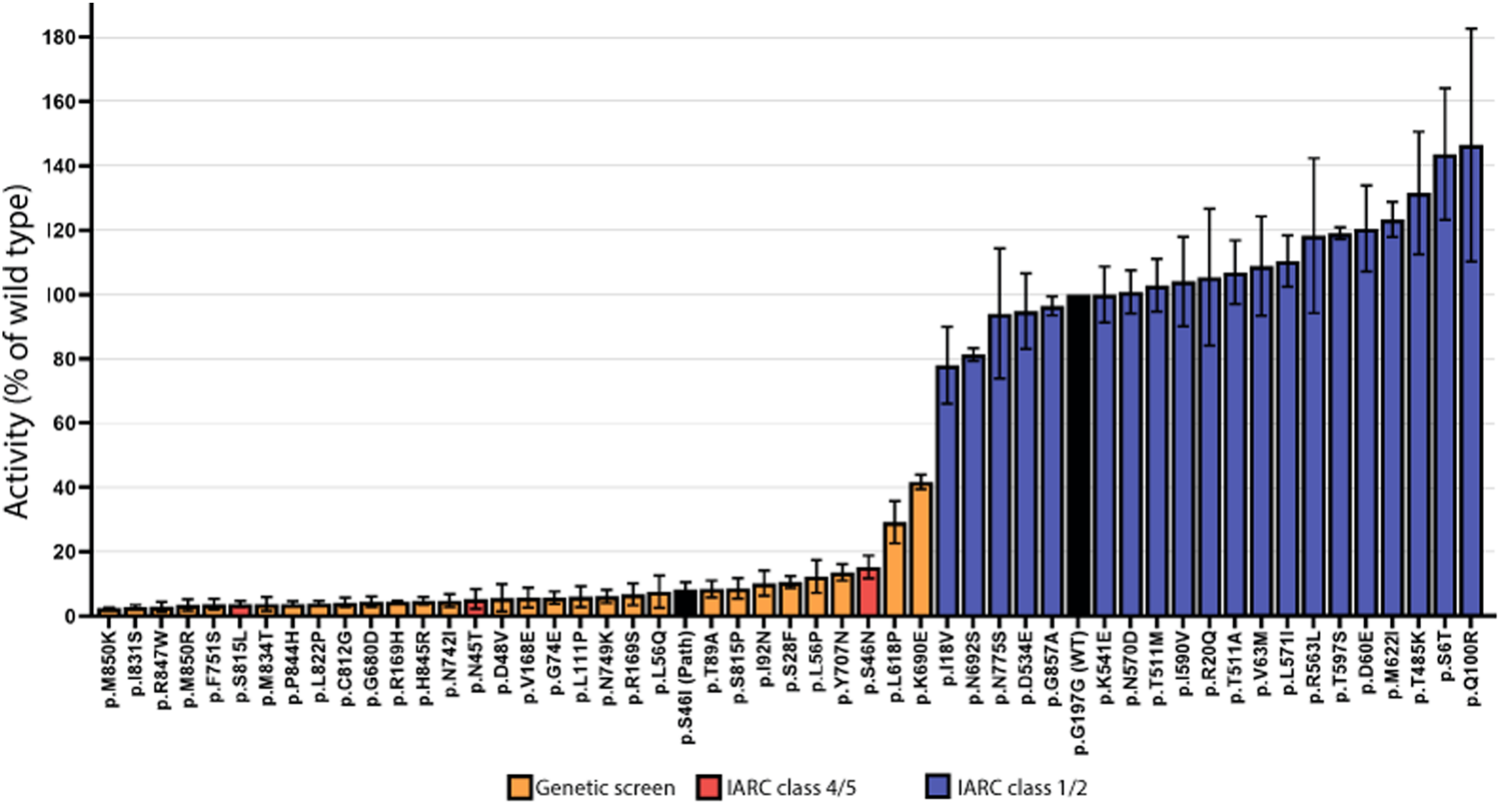
Mismatch repair activity in the complete in vitro mismatch repair activity (CIMRA) assay of PMS2 variants used for calibration and validation of the assay. Relative repair efficiencies for human PMS2 variants used in the calibration and validation of the CIMRA assay. Variants are colored according to their ClinVar classification and their identification as inactivating in the genetic screen. The PMS2 p.S46I variant was included in every assay as a mismatch repair‐deficient pathogenic control and p.G197G as a wild‐type control (Borràs et al., 2013) and are colored black. Bars represent ± Standarderror of the mean (SEM) of three independent experiments.

**Figure 5:**
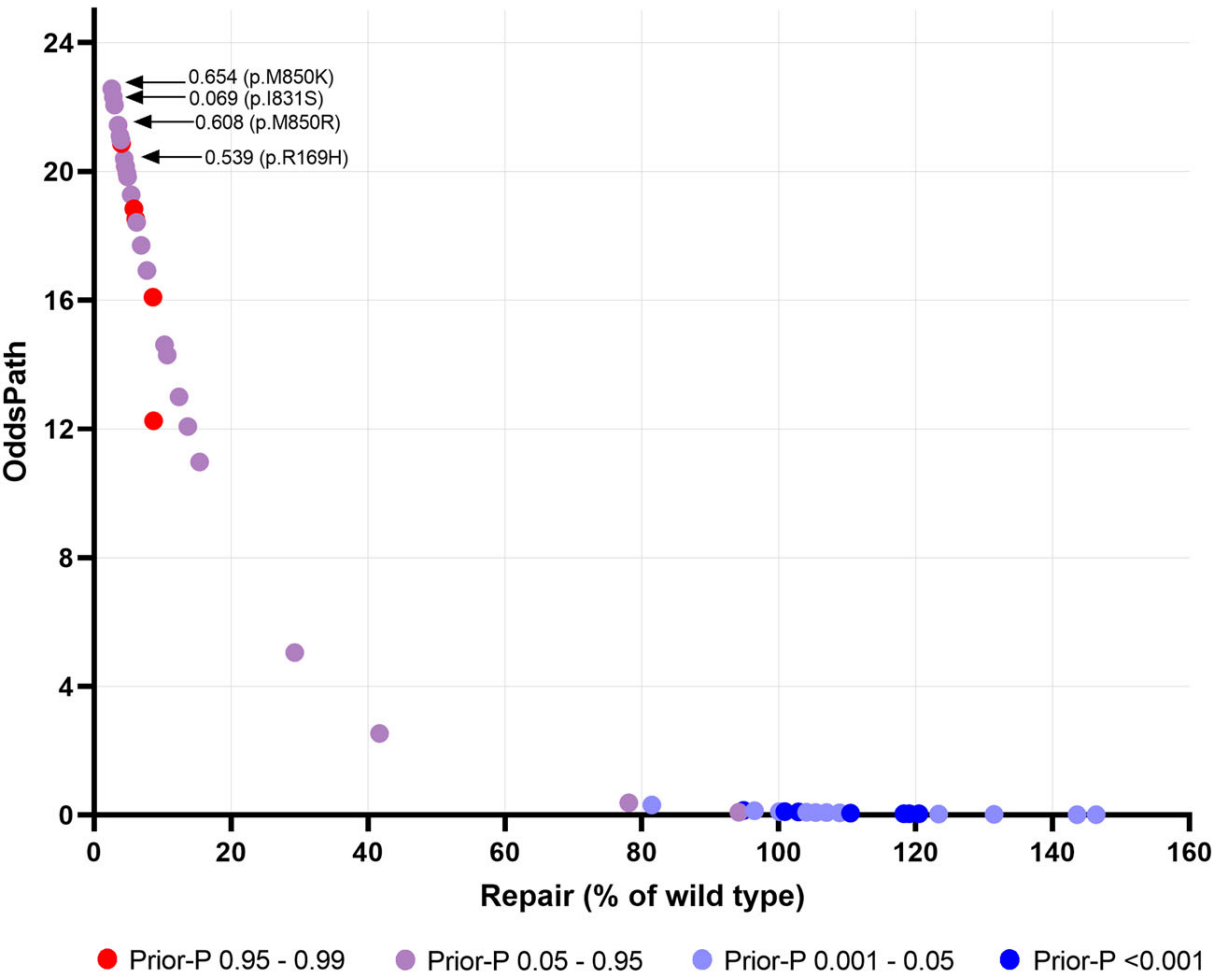
Odds of pathogenicity of PMS2 variants The odds of pathogenicity (OddsPath) for PMS2 variants used in the calibration and validation of the complete in vitro mismatch repair activity (CIMRA) assay plotted against the average relative repair efficiency from the CIMRA assay. The odds of pathogenicity (OddsPath) for each PMS2 variant was derived using the leave‐one‐out procedure. Each variant is colored according to the computational prediction of pathogenicity (Prior‐P) (see Table 1; B. A. Thompson et al., 2013). Four variants for which the bioinformatics‐based Prior‐P and the CIMRA assay‐based OddsPath were strongly discordant are indicated separately in the figure.

We used the CIMRA assay to determine the MMR activity of the set of 51 human PMS2 substitution variants, comprising the 20 class 1/2 variants and 31 (likely) predisposing or genetic screen‐derived inactivating variants (Table 1). The CIMRA assay results demonstrated that all clinically classified (likely) benign protein variants had a near‐wild type MMR activity (range: 78%–146% of wild type), indicating that the CIMRA assay is robust in detecting benign variants with no predicted splice defect. (Likely) predisposing and genetic screen‐derived variants, derived from the databases and the genetic screen, all showed low repair of, on average, 8% of wild type (range: 2.6%–42%; Figure 4 and Table 1). Four variants from the genetic screen, despite having associated moderate to low in silico predictions of pathogenicity (Prior‐P), indicative of non‐predisposing, nevertheless displayed loss of MMR function when tested in the CIMRA assay (p. Arg169His, p. Ile831Ser, p. Met850Arg, and p. Met850Lys; Table 1 and Figure 5). The resistance of the corresponding cell lines to 6‐TG combined with the biochemical MMR deficiency strongly suggests that these are indeed predisposing and indicate false‐negativity of the in silico‐derived Prior‐P.

We then performed linear regression of the CIMRA assay repair levels from the 51 PMS2 variants against the given log‐transformed OddsPath to generate a final calibration equation: log_10_(OddsPath) = −0.0237 * CIMRA assay activity + 1.4127. Statistical metrics derived during cross‐validation showed a good performance of the regression model (*R* ^2^ = 0.95 and root mean squared error = 0.29).

For OddsPath estimation for each individual variant, we used a leave‐one‐out procedure to permit probability estimation. For all variants previously classified as (likely) pathogenic/predisposing by clinical criteria or as inactivating and therefore (proxy) pathogenic/predisposing by the genetic screen, the average calculated OddsPath was 17 (range: 2.5−23), overall considered as supporting to strong strength towards pathogenicity for application under ACMG/AMP code PS3 (Supporting Information: Table S3; https://www.insight-group.org/criteria) (Tavtigian et al., 2018). For all clinically classified (likely) benign variants, the average calculated OddsPath was 0.10 (range: 0.01–0.38), overall considered to be supporting to strong evidence against pathogenicity for application under ACMG/AMP code BS3 (Figure 5, Supporting Information: Table S3). Thus, the CIMRA assay‐derived OddsPath displays a bimodal distribution, in support of its high predictive value for predisposing and for benign variants. The OddsPath for individual variants, and relevant ACMG/AMP codes are shown in Table 1.

As summarized in Table 2, none of the “benign” reference set variants were incorrectly assigned a high OddsPath (in favor of pathogenicity), and none of the “predisposing” reference set variants were incorrectly assigned a low OddsPath.

**Table 2:** CIMRA reference set OddsPath categories.

	Reference sets	
CIMRA assay OddsPath category	Benign	Pathogenic
	*N*	*N*
BS3—Strong	7	
BS3—Moderate	11	
BS3—Supporting	2	
PS3—Supporting		1
PS3—Moderate		12
PS3—Strong		18
Total	20	31
Mean	0.10	17
Range	0.01–0.38	2.5–23

Abbreviations: CIMRA, complete in vitro mismatch repair activity; OddsPath, odds of pathogenicity.

## 4 DISCUSSION

Personalized genetics is futile when it cannot be translated into personalized healthcare through the classification of the pathogenicity of genetic variants. Heterozygous functionally inactivating PMS2 variants, when converted to homozygosity by a somatic event, cause a MMR defect, and the resulting spontaneous mutator phenotype predisposes to cancer. Resulting from partial redundancy, PMS2 deficiency is associated with a relatively low cancer penetrance which makes classification using only clinical criteria extremely challenging. Since a biochemical defect in MMR is causative of cancer predisposition and this defect can be measured and quantified, calibrated and validated functional assays may greatly improve the classification of such VUS.

To enable the calibration and validation of the CIMRA assay for PMS2 VUS we have compiled a set of benign and inactivating (i.e., predisposing) PMS2 variants, the latter mostly derived from a genetic screen, and we tested all variants in the CIMRA assay. All PMS2 protein variants derived from the genetic screen, even those with a low, in silico‐calculated, Prior‐P were functionally defective for MMR in the CIMRA assay. This demonstrates that the loss of DNA damage responses (i.e., 6‐TG resistance), as employed as a selective tool in the genetic screen, is functionally intertwined with the loss of MMR. The derived and then validated regression formula converts the relative activity of a PMS2 variant measured in the CIMRA assay into an OddsPath. Our validation of this formula demonstrates that it is fully concordant with PMS2 variant classification based on clinical or phenotypic (genetic screen‐derived) criteria, with a bimodal distribution of the OddsPath between cancer predisposing and benign variants, despite the conservative approach for calibration of PMS2 variants as compared to calibration of the CIMRA assay for variants in the other three MMR genes. This conservative approach is reflected by the less extreme OddsPath values for PMS2 compared to those calculated from previously derived MLH1, MSH2 and MSH6 calibration formulae (Supporting Information: Table S4) (Drost et al., 2019, 2020).

Despite its high predictive values, the CIMRA assay‐based classification tool may not be infallible. For example, in case a variant retains high MMR activity (resulting in a low OddsPath), some caution must be exercised since (1) the assay is cell‐free and, therefore, some in vivo‐specific defects, such as aberrant intracellular compartmentalization cannot be measured, and (2) the cDNA‐based nature of the assay complicates the interpretation of variants that are predisposing resulting from 5' and 3' UTR and variants that impact splicing. For this reason, known splice variants were excluded from the calibration and the validation of the PMS2 CIMRA assay. Nevertheless, previous data have demonstrated that many MSH2, MSH6, and MLH1 variants at exonic splice sites that, simultaneously, cause an inactivating amino acid substitution can correctly be classified using the CIMRA assay, since the amino acid substitution frequently inactivates protein function (B. A. Thompson et al., 2020). Nevertheless, to further improve the classification of predicted splice‐impacting variants, additional functional evidence is warranted, as recommended by Brnich et al. (Brnich et al., 2019). An example of assays that can be used to assess functional consequences of putative splice, 5′ and 3′ UTR, and cellular compartmentalisation‐perturbed variants are cellular assays that are based on the introduction of the VUS at its genomic locus and testing MSI and loss of toxicity of 6‐TG or methylating agents (Houlleberghs et al., 2016, 2020; Rath et al., 2019). Of further relevance, the incidence of cancer in individuals carrying a fully inactivating PMS2 variant is lower than that in individuals carrying a fully inactivating MSH2 or MLH1 variant, which results from the partial genetic redundancy of PMS2 with minor MMR proteins (notably MLH3 or PMS1). Nevertheless, such a PMS2 variant will still be predisposing.

The high sensitivities and specificities of the CIMRA assay for the classification of VUS in MSH2, MSH6, and MLH1 (Drost et al., 2019, 2020), and now also in PMS2, has led to its inclusion in ACMG/AMP guidelines (https://www.insight-group.org/criteria) as a strong criterion for pathogenicity (PS3) or against pathogenicity (BS3). In these guidelines, the CIMRA assay‐derived OddsPath is defined as strong evidence of pathogenicity (PS3‐strong) when the OddsPath >18.7, a moderate evidence of pathogenicity (PS3‐moderate) with an OddsPath >4.32 and ≤18.7, while an OddsPath of >2.08 and ≤4.32 is considered supportive of pathogenicity (PS3‐supportive) (see also Table 1 and Supporting Information: Table S3). Conversely, a CIMRA assay‐derived OddsPath of ≤0.053 is considered strong evidence against pathogenicity (BS3‐strong), while an OddsPath between 0.053 and 0.48 is considered BS3‐moderate and an OddsPath of >0.23 and ≤0.48 is considered BS3‐supportive.

Currently, functional assays, including the calibrated CIMRA assay, are successfully being integrated with clinical data in the classification of MMR gene VUS in all four MMR proteins, in the framework of the Dutch INVUSE consortium (https://www.icrpartnership.org/project/funding-details/294635). We anticipate that the upcoming ACMG/AMP guidelines that include the CIMRA assay as a strong evidence type to classify VUS in all four MMR genes will not only significantly advance their classification worldwide, but also provide a paradigm for the translation of personalized cancer genetics into personalized healthcare.

## CONFLICTS OF INTEREST

The authors declare no conflicts of interest.

## Supporting information

Supporting information.
