# Supplementary material for: Predictive functional assay‐based classification of PMS2 variants in Lynch syndrome
Source: Hum Mutat. 2022 Apr 28;43(9):1249–58. doi: 10.1002/humu.24387 (PMC9545740; doi:10.1002/humu.24387)
Supplement: Supplementary file 1 — Supporting information. [file HUMU-43-1249-s001.docx]

Supplemental material

Figure S1. **Conservation between mouse and human Pms2 substitution mutations identified in the genetic screen**

Mouse MEQTEGVSTECAKAIKPIDGKSVHQICSGQVVLSLSTAVKELIENSVDAGATTIDLRLKD 60

Human MERAESSSTEPAKAIKPIDRKSVHQICSGQVVLSLSTAVKELVENSLDAGATNIDLKLKD 60

** * *** ******** ********************** *** ***** *** ***

Mouse YGVDLIEVSDNGCGVEEENFEGLALKHHTSKIQEFADLTQVETFGFRGEALSSLCALSDV 120

Human YGVDLIEVSDNGCGVEEENFEGLTLKHHTSKIQEFADLTQVETFGFRGEALSSLCALSDV 120

*********************** ************************************

Mouse TISTCHGSASVGTRLVFDHNGKITQKTPYPRPKGTTVSVQHLFYTLPVRYKEFQRNIKKE 180

Human TISTCHASAKVGTRLMFDHNGKIIQKTPYPRPRGTTVSVQQLFSTLPVRHKEFQRNIKKE 180

****** ** ***** ******* ******** ******* ** ***** **********

Mouse YAKMVQVLQAYCIISAGVRVSCTNQLGQGKRQPVVCTSGSSGMKENIGSVFGQKQLQSLI 240

Human YAKMVQVLHAYCIISAGIRVSCTNQLGQGKRQPVVCTGGSPSIKENIGSVFGQKQLQSLI 240

******** ******** ******************* ** *****************

Mouse PFVQLPPSDAVCEEYGLSTSRTPQNLFYVSGFISQCTHGAGRSATDRQFFFINQRPCDPA 300

Human PFVQLPPSDSVCEEYGLSCSDALHNLFYISGFISQCTHGVGRSSTDRQFFFINRRPCDPA 300

********* ******** * **** ********** *** ********* ******

Mouse KVSKLVNEVYHMYNRHQYPFVVLNVSVDSECVDINVTPDKRQILLQEEKLLLAVLKTSLI 360

Human KVCRLVNEVYHMYNRHQYPFVVLNISVDSECVDINVTPDKRQILLQEEKLLLAVLKTSLI 360

** ******************** ***********************************

Mouse GMFDSDANKLNVNQQPLLDVEGNLVKLHTAELEKPVPGKQDNSPSLKSTADEKRVASISR 420

Human GMFDSDVNKLNVSQQPLLDVEGNLIKMHAADLEKPMVEKQDQSPSLRTG-EEKKDVSISR 419

****** ***** *********** * * * **** *** **** ** ****

Mouse LREAFSLHPTKEIKSRGPETAELTRSFPSEKRGVLSSYPSDVISYRGLRGSQDKLVSPTD 480

Human LREAFSLRHTTENKPHSPKTPEPRRSPLGQKRGMLSSSTSGAISDKGVLRPQKEAVSSSH 479

******* * * * * * * ** *** *** * ** * * **

Mouse SPGDCMDREKIEKDSGLSSTSAGSEEGFSTPEVASSFSSDYNVSSPEDRPSQETINCGDL 540

Human GPSDPTDRAEVEKDSGHGSTSVD-SEGFSIPDTGSHCSSEYAASSPGDRGSQEHVDSQEK 538

* * ** ***** *** **** * * ** * *** ** ***

Mouse DCRPPG----TGQSLKPEDHGYQCKALPL-ARLSPTNAKRFKTEERPSNVNISQRLPGPQ 595

Human APKTDDSFSDVDCHSNQEDTGCKFRVLPQPTNLATPNTKRFKKEEILSSSDICQKLVNTQ 598

** * ** * * **** ** * * * * *

Mouse STSAAEVDVAIKMNKRIVLLEFSLSSLAKRMKQLQHLKAQNKHELSYRKFRAKICPGENQ 655

Human DMSASQVDVAVKINKKVVPLDFSMSSLAKRIKQLHHEAQQSEGEQNYRKFRAKICPGENQ 658

** **** * ** * * ** ****** *** * * * **************

Mouse AAEDELRKEISKSMFAEMEILGQFNLGFIVTKLKEDLFLVDQHAADEKYNFEMLQQHTVL 715

Human AAEDELRKEISKTMFAEMEIIGQFNLGFIITKLNEDIFIVDQHATDEKYNFEMLQQHTVL 718

************ ******* ******** *** ** * ***** ***************

Mouse QAQRLITPQTLNLTAVNEAVLIENLEIFRKNGFDFVIDEDAPVTERAKLISLPTSKNWTF 775

Human QGQRLIAPQTLNLTAVNEAVLIENLEIFRKNGFDFVIDENAPVTERAKLISLPTSKNWTF 778

* **** ********************************:********************

Mouse GPQDIDELIFMLSDSPGVMCRPSRVRQMFASRACRKSVMIGTALNASEMKKLITHMGEMD 835

Human GPQDVDELIFMLSDSPGVMCRPSRVKQMFASRACRKSVMIGTALNTSEMKKLITHMGEMD 838

**** ******************** ******************* **************

Mouse HPWNCPHGRPTMRHVANLDVISQN 859

Human HPWNCPHGRPTMRHIANLGVISQN 862

************** *** *****

The mouse Pms2 protein sequence (NP_032912.2) was aligned to the human PMS2 protein sequence (NP_000526.2) using Clustal Omega (<https://www.ebi.ac.uk/Tools/msa/clustalo/>). Conserved residues that are mutated in the genetic screen for inactivating variants are highlighted in red. An inactivating substitution of a non-conserved triplet encoding a conserved amino acid (Table S2) is highlighted in orange and non-conserved residues are highlighted in green. Asterisks denote conserved residues.

Table S1. **Clinically classified substitution variants in PMS2 derived from the ClinVar/InSiGHT databases**

| **Variant** | | | **Classification** | | |  |
| --- | --- | --- | --- | --- | --- | --- |
| **cDNA^a^** | **Protein^b^** | **ClinVar ID^c^** | **Odds for Causality^d^** | **ClinVar Interpretation^e^** | **IARC Class (InSiGHT)^f^** | **Meets inclusion criteria?** |
| c.3G>A | p.Met1Ile | 444148 | NA | P | NA | N |
| c.2T>G | p.Met1Arg | 233615 | NA | P | NA | N |
| c.2T>A | p.Met1Lys | 182809 | NA | P | NA | N |
| c.1A>T | p.Met1Leu | 152491 | NA | P | NA | N |
| c.1A>G | p.Met1Val | 96798 | 1.003 | LP* | 4 | N |
| c.17G>C | p.Ser6Thr | 138704 | NA | B | NA | Y |
| c.52A>G | p.Ile18Val | 41713 | 0.062 | LB^*^ | 2 | Y |
| c.59G>A | p.Arg20Gln | 41716 | NA | B^*^ | 1 | Y |
| c.134A>C | p.Asn45Thr | 434024 | NA | LP | NA | Y |
| c.137G>T | p.Ser46Ile | 9245 | 3.083 | LP^*^ | 4 | N |
| c.137G>A | p.Ser46Asn | 91301 | NA | LP | NA | Y |
| c.180C>G | p.Asp60Glu | 91315 | 0.164 | LB^*^ | 2 | Y |
| c.187G>A | p.Val63Met | 549723 | NA | LB | NA | Y |
| c.299A>G | p.Gln100Arg | 434026 | NA | B | NA | Y |
| c.903G>T | p.Lys301Asn | 96852 | NA | P* | 4 | N |
| c.1454C>A | p.Thr485Lys | 41702 | NA | B^*^ | 1 | Y |
| c.1531A>G | p.Thr511Ala | 41703 | NA | B^*^ | 1 | Y |
| c.1532C>T | p.Thr511Met | 41704 | NA | B^*^ | 1 | Y |
| c.1602C>G | p.Asp534Glu | 484280 | NA | LB | NA | Y |
| c.1621A>G | p.Lys541Glu | 135065 | NA | B | NA | Y |
| c.1688G>T | p.Arg563Leu | 41705 | 0.014 | LB^*^ | 2 | Y |
| c.1708A>G | p.Asn570Asp | 135066 | NA | LB | NA | Y |
| c.1711C>A | p.Leu571Ile | 41706 | NA | LB^*^ | 2 | Y |
| c.1768A>G | p.Ile590Val | 484249 | NA | LB | NA | Y |
| c.1789A>T | p.Thr597Ser | 41707 | 0.135 | B^*^ | 1 | Y |
| c.1866G>A | p.Met622Ile | 41708 | NA | B^*^ | 1 | Y |
| c.2002A>G | p.Ile668Val | 190109 | NA | LP | NA | N |
| c.2075A>G | p.Asn692Ser | 619585 | NA | LB | NA | Y |
| c.2324A>G | p.Asn775Ser | 36689 | NA | B^*^ | 1 | Y |
| c.2444C>T | p.Ser815Leu | 91343 | 1.825 | LP^*^ | 4 | Y |
| c.2570G>C | p.Gly857Ala | 36691 | NA | B^*^ | 1 | Y |

^a^ Nucleotide numbering reflects cDNA numbering with +1 corresponding to the A of the translation initiation codon in the GenBank reference sequence. *PMS2*: GenBank NM_000535.7.

^b^ Amino acid numbering is based on the cDNA with +1 corresponding to the translation initiation codon in the GenBank reference sequence. PMS2: NP_000526.2

^c^ ClinVar Variation ID, see <https://www.ncbi.nlm.nih.gov/clinvar/>

^d^ Odds for causality as classified by the InSiGHT Variant Interpretation Committee (Thompson et al., 2014). NA = not available

^e^ ClinVar Variant Interpretation based on ACMG/AMP recommendations for Mendelian disorders. B = Benign, LB = Likely Benign, LP = Likely Pathogenic. Variants reviewed by an expert panel are denoted by ^*^.

^f^ IARC classification for each variant as classified by the InSiGHT Variant Interpretation Committee (Thompson et al., 2014). NA = not available.

**Table S2. Overview of inactivating substitution variants identified in the genetic screen**

| **Variant** | | |  |  |
| --- | --- | --- | --- | --- |
| **cDNA (mouse)^b^** | **Mouse allele^a^** | **Human allele^c^** | ***In silico* Prior-P^d^** | **Meets inclusion criteria?** |
| c.83C>T | p.Ser28Phe | p.Ser28Phe | 0.881 | Y |
| c.143A>T | p.Asp48Val | p.Asp48Val | 0.891 | Y |
| c.167T>C | p.Leu56Pro | p.Leu56Pro | 0.829 | Y |
| c.167T>A | p.Leu56Gln | p.Leu56Gln | 0.766 | Y |
| c.221G>A | p.Gly74Glu | p.Gly74Glu | 0.959 | Y |
| c.265A>G | p.Thr89Ala | p.Thr89Ala | 0.965 | Y |
| c.275T>A | p.Ile92Asn | p.Ile92Asn | 0.901 | Y |
| c.332T>C | p.Leu111Pro | p.Leu111Pro | 0.961 | Y |
| c.341T>A | p.Leu114Gln^f^ | p.Leu114His | N/A | N |
| c.503T>A | p.Val168Glu | p.Val168Glu | 0.949 | Y |
| c.505C>A | p.Arg169Ser | p.Arg169Ser | 0.878 | Y |
| c.506G>A | p.Arg169His | p.Arg169His | 0.539 | y |
| c.1844T>C | p.Leu615Pro | p.Leu618Pro | 0.708 | Y |
| c.2030G>A | p.Gly677Asp | p.Gly680Asp | 0.960 | Y |
| c.2059A>G | p.Lys687Glu | p.Lys690Glu | 0.862 | Y |
| c.2075T>C | p.Leu692Pro^e^ | p.Ile695Pro | N/A | N |
| c.2081T>C | p.Leu694Pro^e^ | p.Ile697Pro | N/A | N |
| c.2110T>A | p.Tyr704Asn | p.Tyr707Asn | 0.832 | Y |
| c.2216A>T | p.Asn739Ile | p.Asn742Ile | 0.940 | Y |
| c.2238T>A | p.Asn746Lys | p.Asn749Lys | 0.916 | Y |
| c.2243T>C | p.Phe748Ser | p.Phe751Ser | 0.857 | Y |
| c.2425T>G | p.Cys809Gly | p.Cys812Gly | 0.780 | Y |
| c.2434T>C | p.Ser812Pro | p.Ser815Pro | 0.967 | Y |
| c.2456T>C | p.Leu819Pro | p.Leu822Pro | 0.961 | Y |
| c.2483T>G | p.Ile828Ser | p.Ile831Ser | 0.069 | y |
| c.2492T>C | p.Met831Thr | p.Met834Thr | 0.918 | Y |
| c.2521C>A | p.Pro841His | p.Pro844His | 0.854 | Y |
| c.2525A>G | p.His842Arg | p.His845Arg | 0.946 | Y |
| c.2530A>T | p.Arg844Trp | p.Arg847Trp | 0.891 | Y |
| c.2540T>G | p.Met847Arg | p.Met850Arg | 0.608 | Y |
| c.2540T>A | p.Met847Lys | p.Met850Lys | 0.654 | Y |

^a^ Amino acid position is based on the Pms2 GenBank reference sequence NP_032912.2 with +1 corresponding to the translation initiation codon

^b^ Nucleotide numbering reflects cDNA numbering with +1 corresponding to the A of the translation initiation codon in the mouse Pms2 GenBank reference sequence NM_008886.2.

^c^ Amino Acid position is based on the Human PMS2 GenBank reference sequence NP_000526.2 with +1 corresponding to the translation initiation codon. Equivilent Human residue was determined from an alignment of mouse *Pms2*  and human *PMS2* cDNA (Figure S1)

^d^ *In silico* prior probabilities as previously calculated (B. A. Thompson et al., 2013)

^e^Substitution at non-conserved residue

^f^Substitution with an unavailable Prior-P

**Table S3. ClinGen InSiGHT Hereditary Colorectal Cancer/Polyposis Variant Curation Expert Panel Specifications to the ACMG/AMP Variant Interpretation Guidelines**

| **ACMG Criteria^a^** | | **OddsPath^b^** | **OddsPath^c^** |
| --- | --- | --- | --- |
| **Evidence of pathogenicity (PS3)** | Strong | >18.7 | >18.7 |
|  | Moderate | >4.3 and ≤18.7 | >4.3 and ≤18.7 |
|  | Supporting | >2.08 and ≤4.3 | >2.08 and ≤4.3 |
| **Evidence of neutrality (BS3)** | Supporting | >0.23 and ≤0.48 | >0.05 and ≤0.48 |
|  | Moderate | ≥0.053 and ≤0.23 | NA |
|  | Strong | ≤0.053 | ≤0.05 |

^a^ Criteria for variant classification as defined by the ACMG/AMP (Richards et al., 2015)

^b^ Quantitative modelling of the ACMG classification thresholds (Tavtigian et al., 2018)

^c^ OddsPath thresholds for the revised ACMG Criteria <https://www.insight-group.org/criteria>

**Table S4. CIMRA assay activities for variants in all MMR genes, converted to OddsPath by using the respective calibration formulae**

|  | **MMR protein OddsPath^±^** | | | |
| --- | --- | --- | --- | --- |
| **CIMRA assay**  **(% repair of WT)** | **MSH2** | **MSH6** | **PMS2** | **MLH1** |
| 1 | 276.376 | 65.330 | 24.491 | 276.376 |
| 2 | 243.781 | 60.921 | 23.190 | 243.781 |
| 3 | 215.030 | 56.809 | 21.958 | 215.030 |
| 4 | 189.671 | 52.974 | 20.792 | 189.671 |
| 5 | 167.302 | 49.399 | 19.688 | 167.302 |
| 6 | 147.571 | 46.064 | 18.642 | 147.571 |
| 7 | 130.167 | 42.955 | 17.652 | 130.167 |
| 8 | 114.815 | 40.056 | 16.715 | 114.815 |
| 9 | 101.274 | 37.352 | 15.827 | 101.274 |
| 10 | 89.331 | 34.831 | 14.986 | 89.331 |
| 11 | 78.795 | 32.480 | 14.191 | 78.795 |
| 12 | 69.502 | 30.288 | 13.437 | 69.502 |
| 13 | 61.306 | 28.243 | 12.723 | 61.306 |
| 14 | 54.075 | 26.337 | 12.048 | 54.075 |
| 15 | 47.698 | 24.559 | 11.408 | 47.698 |
| 16 | 42.073 | 22.902 | 10.802 | 42.073 |
| 17 | 37.111 | 21.356 | 10.228 | 37.111 |
| 18 | 32.734 | 19.914 | 9.685 | 32.734 |
| 19 | 28.874 | 18.570 | 9.171 | 28.874 |
| 20 | 25.468 | 17.317 | 8.684 | 25.468 |
| 21 | 22.465 | 16.148 | 8.222 | 22.465 |
| 22 | 19.815 | 15.058 | 7.786 | 19.815 |
| 23 | 17.478 | 14.042 | 7.372 | 17.478 |
| 24 | 15.417 | 13.094 | 6.981 | 15.417 |
| 25 | 13.599 | 12.210 | 6.610 | 13.599 |
| 26 | 11.995 | 11.386 | 6.259 | 11.995 |
| 27 | 10.580 | 10.617 | 5.927 | 10.580 |
| 28 | 9.333 | 9.901 | 5.612 | 9.333 |
| 29 | 8.232 | 9.232 | 5.314 | 8.232 |
| 30 | 7.261 | 8.609 | 5.032 | 7.261 |
| 31 | 6.405 | 8.028 | 4.764 | 6.405 |
| 32 | 5.649 | 7.486 | 4.511 | 5.649 |
| 33 | 4.983 | 6.981 | 4.272 | 4.983 |
| 34 | 4.395 | 6.510 | 4.045 | 4.395 |
| 35 | 3.877 | 6.070 | 3.830 | 3.877 |
| 36 | 3.420 | 5.661 | 3.627 | 3.420 |
| 37 | 3.016 | 5.279 | 3.434 | 3.016 |
| 38 | 2.661 | 4.922 | 3.252 | 2.661 |
| 39 | 2.347 | 4.590 | 3.079 | 2.347 |
| 40 | 2.070 | 4.280 | 2.915 | 2.070 |
| 41 | 1.826 | 3.991 | 2.761 | 1.826 |
| 42 | 1.611 | 3.722 | 2.614 | 1.611 |
| 43 | 1.421 | 3.471 | 2.475 | 1.421 |
| 44 | 1.253 | 3.236 | 2.344 | 1.253 |
| 45 | 1.105 | 3.018 | 2.219 | 1.105 |
| 46 | 0.975 | 2.814 | 2.101 | 0.975 |
| 47 | 0.860 | 2.624 | 1.990 | 0.860 |
| 48 | 0.759 | 2.447 | 1.884 | 0.759 |
| 49 | 0.669 | 2.282 | 1.784 | 0.669 |
| 50 | 0.590 | 2.128 | 1.689 | 0.590 |
| 51 | 0.521 | 1.984 | 1.600 | 0.521 |
| 52 | 0.459 | 1.850 | 1.515 | 0.459 |
| 53 | 0.405 | 1.726 | 1.434 | 0.405 |
| 54 | 0.357 | 1.609 | 1.358 | 0.357 |
| 55 | 0.315 | 1.500 | 1.286 | 0.315 |
| 56 | 0.278 | 1.399 | 1.218 | 0.278 |
| 57 | 0.245 | 1.305 | 1.153 | 0.245 |
| 58 | 0.216 | 1.217 | 1.092 | 0.216 |
| 59 | 0.191 | 1.135 | 1.034 | 0.191 |
| 60 | 0.168 | 1.058 | 0.979 | 0.168 |
| 61 | 0.148 | 0.987 | 0.927 | 0.148 |
| 62 | 0.131 | 0.920 | 0.878 | 0.131 |
| 63 | 0.115 | 0.858 | 0.831 | 0.115 |
| 64 | 0.102 | 0.800 | 0.787 | 0.102 |
| 65 | 0.090 | 0.746 | 0.745 | 0.090 |
| 66 | 0.079 | 0.696 | 0.706 | 0.079 |
| 67 | 0.070 | 0.649 | 0.668 | 0.070 |
| 68 | 0.062 | 0.605 | 0.633 | 0.062 |
| 69 | 0.054 | 0.564 | 0.599 | 0.054 |
| 70 | 0.048 | 0.526 | 0.567 | 0.048 |
| 71 | 0.042 | 0.490 | 0.537 | 0.042 |
| 72 | 0.037 | 0.457 | 0.509 | 0.037 |
| 73 | 0.033 | 0.426 | 0.482 | 0.033 |
| 74 | 0.029 | 0.398 | 0.456 | 0.029 |
| 75 | 0.026 | 0.371 | 0.432 | 0.026 |
| 76 | 0.023 | 0.346 | 0.409 | 0.023 |
| 77 | 0.020 | 0.322 | 0.387 | 0.020 |
| 78 | 0.018 | 0.301 | 0.367 | 0.018 |
| 79 | 0.016 | 0.280 | 0.347 | 0.016 |
| 80 | 0.014 | 0.261 | 0.329 | 0.014 |
| 81 | 0.012 | 0.244 | 0.311 | 0.012 |
| 82 | 0.011 | 0.227 | 0.295 | 0.011 |
| 83 | 0.009 | 0.212 | 0.279 | 0.009 |
| 84 | 0.008 | 0.198 | 0.264 | 0.008 |
| 85 | 0.007 | 0.184 | 0.250 | 0.007 |
| 86 | 0.006 | 0.172 | 0.237 | 0.006 |
| 87 | 0.006 | 0.160 | 0.224 | 0.006 |
| 88 | 0.005 | 0.150 | 0.212 | 0.005 |
| 89 | 0.004 | 0.139 | 0.201 | 0.004 |
| 90 | 0.004 | 0.130 | 0.190 | 0.004 |
| 91 | 0.003 | 0.121 | 0.180 | 0.003 |
| 92 | 0.003 | 0.113 | 0.171 | 0.003 |
| 93 | 0.003 | 0.105 | 0.162 | 0.003 |
| 94 | 0.002 | 0.098 | 0.153 | 0.002 |
| 95 | 0.002 | 0.092 | 0.145 | 0.002 |
| 96 | 0.002 | 0.085 | 0.137 | 0.002 |
| 97 | 0.002 | 0.080 | 0.130 | 0.002 |
| 98 | 0.001 | 0.074 | 0.123 | 0.001 |
| 99 | 0.001 | 0.069 | 0.117 | 0.001 |
| 100 | 0.001 | 0.065 | 0.110 | 0.001 |

^±^OddsPath values for each CIMRA repair percentage based on the following formulae:

- PMS2: (OddsPath) = 10^((-0.0237*CIMRA assay activity) + 1.4127)
- MLH1 and MSH2: (OddsPath) = 10^((-0.0545*CIMRA assay activity) + 2.496)
- MSH6: (OddsPath) = 10^((-0.03035*CIMRA assay activity) + 1.8455)
